# Responsive Proteins in Wheat Cultivars with Contrasting Nitrogen Efficiencies under the Combined Stress of High Temperature and Low Nitrogen

**DOI:** 10.3390/genes8120356

**Published:** 2017-11-29

**Authors:** Peerzada Yasir Yousuf, Elsayed Fathi Abd_Allah, Mohd Nauman, Ambreen Asif, Abeer Hashem, Abdulaziz A. Alqarawi, Altaf Ahmad

**Affiliations:** 1 Department of Botany, Jamia Hamdard, New Delhi 110062, India; syedyasar55@gmail.com (P.Y.Y.); mnaumanq@gmail.com (M.N.); 2 Plant Production Department, College of Food and Agricultural Sciences, King Saud University, P.O. Box. 2460, Riyadh 11451, Saudi Arabia; eabdallah@ksu.edu.sa (E.F.A.); alqarawi@ksu.edu.sa (A.A.A.); 3 Department of Botany, Aligarh Muslim University, Aligarh 251002, India; ambreenasif1@gmail.com; 4 Botany and Microbiology Department, College of Science, King Saud University, P.O. Box. 2460, Riyadh 11451, Saudi Arabia; habeer@ksu.edu.sa

**Keywords:** proteomics, wheat, nitrogen, nitrogen efficiency, high temperature

## Abstract

Productivity of wheat (*Triticum aestivum*) is markedly affected by high temperature and nitrogen deficiency. Identifying the functional proteins produced in response to these multiple stresses acting in a coordinated manner can help in developing tolerance in the crop. In this study, two wheat cultivars with contrasting nitrogen efficiencies (N-efficient VL616 and N-inefficient UP2382) were grown in control conditions, and under a combined stress of high temperature (32 °C) and low nitrogen (4 mM), and their leaf proteins were analysed in order to identify the responsive proteins. Two-dimensional electrophoresis unravelled sixty-one proteins, which varied in their expression in wheat, and were homologous to known functional proteins involved in biosynthesis, carbohydrate metabolism, energy metabolism, photosynthesis, protein folding, transcription, signalling, oxidative stress, water stress, lipid metabolism, heat stress tolerance, nitrogen metabolism, and protein synthesis. When exposed to high temperature in combination with low nitrogen, wheat plants altered their protein expression as an adaptive means to maintain growth. This response varied with cultivars. Nitrogen-efficient cultivars showed a higher potential of redox homeostasis, protein stability, osmoprotection, and regulation of nitrogen levels. The identified stress-responsive proteins can pave the way for enhancing the multiple-stress tolerance in wheat and developing a better understanding of its mechanism.

## 1. Introduction

The burgeoning global population, on one hand, and a decrease in plant productivity, on the other, have led the world to a situation of food crisis [1]. Anthropogenic turbulences in the environment are the main setbacks to the global food production, among which elevated temperature deserves special mention [2,3,4]. Atmospheric temperature has been estimated to rise by 0.2 °C per decade, attaining a rise of 1.8–4.0 °C by the end of this century [5]. As per the recent report of Intergovernmental Panel on Climate Change, a temperature rise of only 1 °C above pre-industrial era is likely to have a negative impact on the yield of major crops (wheat, rice, and maize) in both tropical and temperate regions [6]. It is estimated that Indian lowlands, that produce almost 15% of global production, will change into heat-stressed and short-season production places [7].

Deficiency of inorganic nitrogen is another key limiting factor in agricultural productivity. The increased crop productivity has been associated with a 20-fold increase in the global use of nitrogen (N) fertilizer during the past five decades [8], and this is expected to increase by 3-fold by the year 2050 [9]. 

Although increase in cereal productivity has matched population growth during the past years, it is a matter of concern how the predicted environmental conditions will affect crop production in future [10,11]. The problems associated with plant productivity are due to multiple stresses acting in a coordinated manner. Studies have focused largely on response mechanisms of plants to single-stress conditions [12,13], and the combinational effects of different stresses are still poorly understood.

Stress-responsive genes are often identified by expression profiling, following exposure to high level of stresses, leading to identification of numerous signalling components and downstream effectors [14,15,16,17]. Abiotic stress experiments have identified processes, and genes involved in plant survival under extreme conditions [18,19]. Despite these successes, there are few examples of extension of such research to crop species [20,21]. 

Wheat is the most widely grown crop of the world in terms of the total harvested area [22], and currently fulfils about 20 per cent of the human calorie consumption [23]. Production of wheat is affected markedly by high temperature [24,25,26], and low nitrogen [27]. Asseng et al. [28] reported about 6% decrease in global wheat production for each degree of temperature increase. Elevated temperature alters uptake and allocation of N, thus intensifying N deficiency in plants [29]. Nitrogen stress also restricts biomass accumulation and overall growth in wheat [30]. It is therefore desirable to examine the response of wheat plants to the combined stress at different levels, in order to develop multiple-stress-tolerant smart plants This study is focused on proteins expressed in wheat cultivars exposed to a combined stress of high temperature and low nitrogen, with a hope that it will help in determining a regulatory network and developing tolerance to stress conditions in this crop.

## 2. Materials and Methods

### 2.1. Plant Culture and Treatments

**Figure 1:**
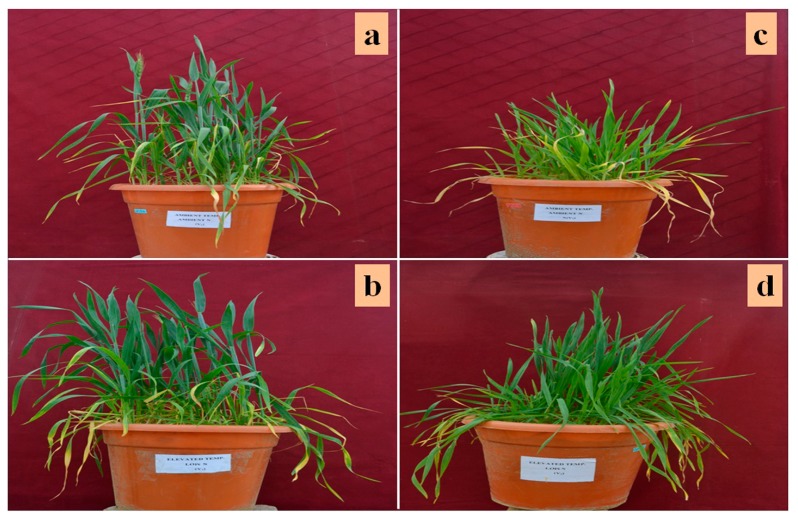
Plants of wheat cultivar VL616 grown under (**a**) control, and (**b**) treatment conditions, and cultivar UP2382 grown under (**c**) control, and (**d**) treatment conditions at the time of sampling.

In an earlier experiment, N-efficient and N-inefficient genotypes of wheat were identified on the basis of physiological and biochemical analyses [31]. Healthy and authenticated seeds of the N-efficient (*Triticum aestivum* cv. VL616) and N-inefficient (*T. aestivum* cv. UP2382) wheat cultivars were procured from Vivekanand Parvatiya Krishi Anusandhan Sansthan (Almora, India) and Gobind Ballabh University of Agriculture and Technology (Pantnagar, India), respectively. These were surface-sterilized with 0.1% mercuric chloride for 1–3 min, rinsed thoroughly with distilled water, and germinated in plastic pots filled with soil up to 7 cm below their mouth. Plants were grown in pots in open top chambers at Indian Agricultural Research Institute, New Delhi, India. Nine pots were kept in each chamber with 10 plants in every pot. A control set of potted plants was provided with an optimum level of N (10 mM) and ambient temperature (26 °C), while the treatment set was grown under conditions of low N levels (4 mM) and high temperature (32 °C). The temperature was regulated by infrared heating tubes. Sampling of leaf with three biological and thee technical replicates was done in morning hours at 45-day growth stage (Figure 1). The sampled leaves were immediately dipped in liquid nitrogen and stored at −80 °C until proteomic analysis. 

### 2.2. Protein Extraction

Proteins were extracted from leaf samples by the phenol method of Isaacson et al. [32]. Two grams of leaf material was ground to fine powder in liquid nitrogen and suspended in 10 mL of extraction buffer containing 50 mM HEPES [4-(2-hydroxyethyl)-1-piperazineethanesulfonic acid], 2% β-mercaptoethanol, 700 mM sucrose, 1 mM PMSF (Phenylmethanesulfonyl fluoride), 50 mM EDTA (Ethylenediaminetetraacetic acid) and 100 mM KCl, with pH adjusted to 7.5. Fifteen millilitres of phenol was added, and the solution mixed in a cold room rocker for 30 min. The solution was then subjected to centrifugation at 3000× *g* for 10 min at 4 °C. The top phenolic phase was carefully recovered in a separate tube, and incubated at −20 °C overnight for precipitation after adding 15 mL of ice-cold 0.1 M ammonium acetate solution. Proteins were pelleted by centrifuging at 6000× *g* for 15 min at 4 °C, and the pellet washed first by methanol, then two times with acetone. The resultant pellet was centrifuged at 3000× *g* after each washing step, then dried and solubilised in buffer containing 2 M thiourea, 7 M urea, 4% CHAPS (3-[(3-Cholamidopropyl)dimethylammonio]-1-propanesulfonate hydrate), and 50 mM DTT (Dithiothreitol). Proteins in the samples were quantified by the Bradford method, using bovine serum albumin (Sigma-Aldrich, USA) as standard.

### 2.3. Two-Dimensional Gel Electrophoresis

Two-dimensional electrophoresis was carried out following the method of O’Farrel [33]. An immobiline dry strip gel (11 cm, pH 4–7; Bio-Rad Laboratories, Inc., Hercules, CA, USA) was rehydrated at 20 °C for 14 h in 200 µL of sample containing 400 µg protein. Isoelectric focusing was carried out in a isoelectric focusing apparatus (PROTEAN^®^ IEF system, Bio-Rad Laboratories, Inc., Hercules, CA, USA). The voltages applied were 250 V for 1 h, 500 V for 1 h, 1000 V for 2 h, 2000 V for 2 h, linear increase of 8000 V for 18 h and 500 V for 1 h. After the completion of isoelectric focusing, the strips were subjected to reduction by the reduction buffer containing 50 mM Tris (pH 8.8), 8 M urea, 20% glycerol, 2% SDS (Sodium Dodecyl Sulfate) and 130 mM DTT and then alkylated for 15 min by alkylation buffer containing Tris (pH 8.8), 8 M urea, 20% glycerol, 2% SDS and 135 mM iodoacetamide. The SDS-PAGE (Sodium Dodecyl Sulfate Polyacrylamide Gel Electrophoresis) was carried out in vertical large format electrophoresis cell (PROTEAN^®^ Plus Dodeca Cell, Bio-Rad Laboratories, Inc., Hercules, CA, USA) for separation of proteins and focusing, using 12% SDS at constant voltage of 250 V. Gels were stained with Coomassie Brilliant Blue dye, and then destained by washing several times with MilliQ water (Milli-Q^®^ Reference, Merck Millipore, Billerica, MA, USA).

### 2.4. Gel Analysis

Images of the gel were digitised using gel documentation system (GS-900™ Calibrated Densitometer, Bio-Rad Laboratories, Inc., Hercules, CA, USA) for further analysis based on spot density, relative abundance, and location (for pH and mass). The image analysis was performed with image master PDQuest software (version 8.0, Bio-Rad Laboratories, Inc., Hercules, CA, USA). The optimized parameters were as follows: saliency 2.0, partial threshold 4 and minimum area 50. Normalization of each spot value was done in terms of percentage of the total volume of all the gel spots for rectification of unevenness, due to quantitative disparity in spot intensities. Spots were quantified on the basis of their relative volume, which was determined by the ratio of the volume of a single spot to the whole set of spots. Spots with more than 2-fold change in volume during the treatment or with significant variation between the control and other treatments, as determined by the paired Student’s *t*-test (*p* ≤ 0.05), were regarded as the treatment-responsive proteins.

### 2.5. In-Gel Digestion and Protein Identification

Protein spots with more than two-fold change in their intensity were excised from gels and dehydrated with 50 µL of solution, containing acetonitrile and 50 mM ammonium bicarbonate in 2:1 ratio, for 5 min. The dehydrated protein spots were reduced with 15 mM DTT at 60 °C for 1 h and subjected to alkylation by 100 mM isoamyl alcohol in dark for 15 min, rehydrated with 50 mM ammonium bicarbonate and then dried in a speed vac. Dried gel slices were subjected to rehydration with 15 µL of working trypsin (Sequencing grade, Promega, Madison, WI, USA) at 37 °C overnight. Supernatant was taken, and 20% acetonitrile + 1% formic acid were added to the remaining gel slice for further extraction. Final supernatant was dried in a speed vac, until the volume was reduced to 25–50 µL. The final volume was analysed with mass spectrometer (4800 MALDI TOF/TOF™, Applied Biosystems/MDS SCIEX, Foster City, CA, USA), with the peptide tolerance of 150 ppm and peptide charge of 1+. Significant hits, as defined by MASCOT server probability analysis (*p* < 0.05), were accepted. Peptides were searched with NCBInr database, taxonomy of green plants, trypsin of the digestion enzyme, one missed cleavage site, partial modification of cysteine carboamidomethylated and methionine oxidized. NCBI [34] and UniProt [35] databases were used to assemble the functional information of identified proteins. On the other hand, pTARGET and UniProt databases were used to recognize the sub-cellular location of identified proteins [36]. Identified proteins were categorized on the basis of their biological functions.

### 2.6. Statistical Analyses

All experiments in the present study were performed from pool of three biological and three experimental replicates. A two-tailed Student’s *t*-test with significance of 95% was performed on normalised value of protein spots, with the help of SPSS software (SPSS for Windows, Version 16.0. SPSS Inc., Chicago, IL, USA).

## 3. Results

### 3.1. Protein Profiling and Spatial Categorization of Differential Protein Expression

**Figure 2:**
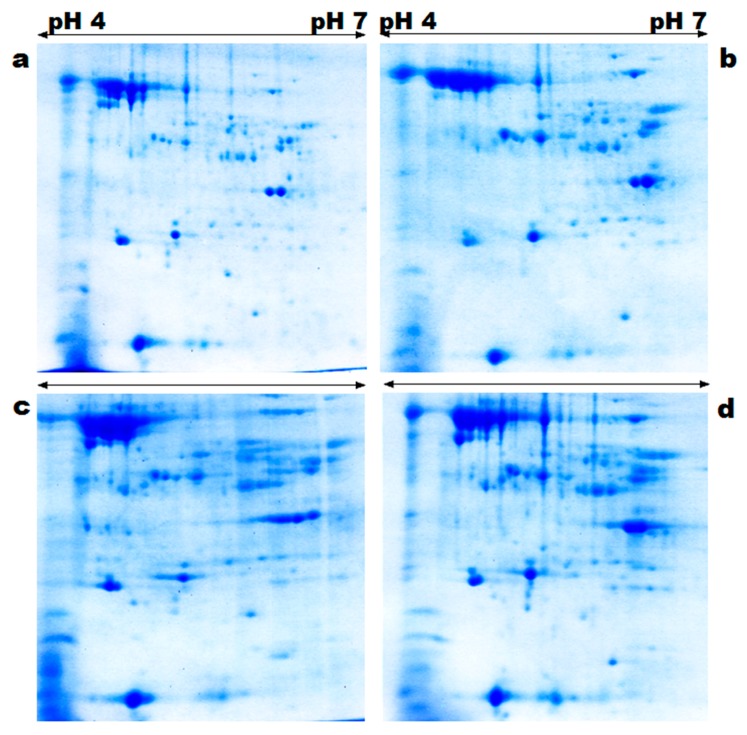
2-DE gel images representing the differentially-expressed leaf proteins of UP2382 (**a**, control; **b**, stress conditions) and VL616 (**c**, control; **d**, stress conditions) cultivars of wheat.

**Figure 3:**
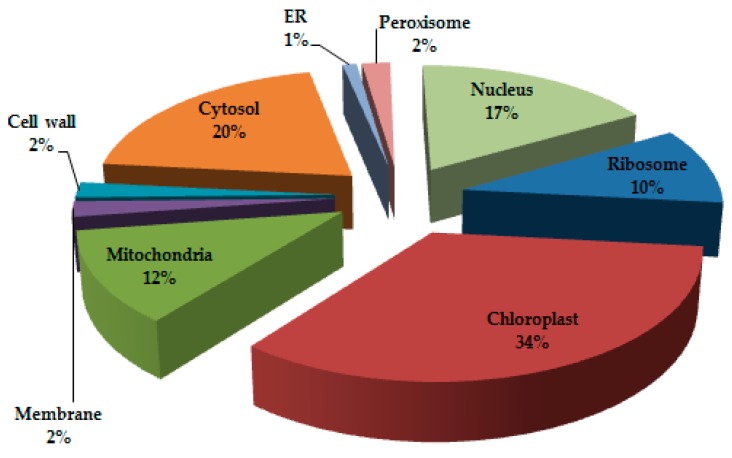
Pie diagram depicting the spatial distribution of differentially-expressed proteins at subcellular level. ER: endoplasmic reticulum.

During two-dimensional gel electrophoresis (2-DE), leaf proteins of the two wheat varieties were distributed throughout the gel. The staining of gels detected 484 protein spots. Of these, 61 (12%) proteins were differentially expressed with more than two-fold change from the control; 42 (69%) of them were upregulated and 19 (31%) downregulated. These differentially expressed proteins, along with their position in 2-DE profiles, are illustrated in Figure 2. They belonged to different subcellular sites (Figure 3); most of them to chloroplast (34%), then to cytosol (20%), nucleus (17%), mitochondrion (12%), and ribosome (10%). A few of them also belonged to peroxisome (2%), membrane (2%), cell wall (2%), and endoplasmic reticulum (ER) (1%).

### 3.2. Functional Cataloguing and Expression-Profile Analysis

**Figure 4:**
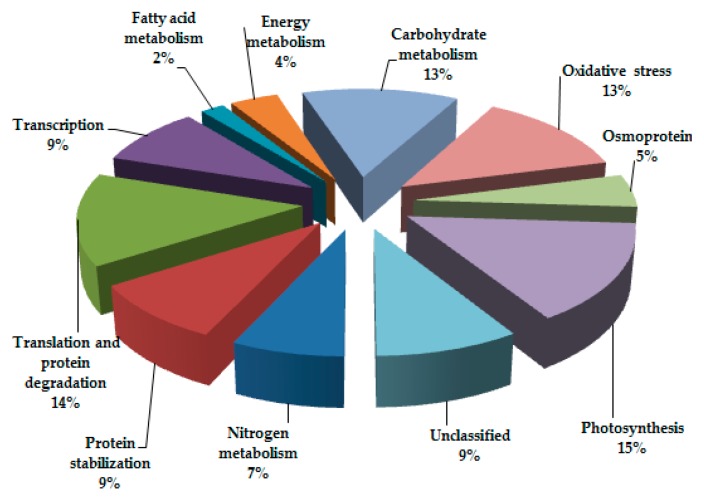
Pie diagram showing the functional cataloguing of differentially-expressed proteins.

Among the 61 identified proteins, 55 (76%) exhibited homology with proteins of known functions, while the rest 6 (24%) were unknown. Based on their association with physiological processes, these proteins were categorized into eleven major groups (Figure 4), viz., nitrogen metabolism (7%), translation and protein degradation (14%), transcription (9%), fatty acid metabolism (2%), energy metabolism (4%), carbohydrate metabolism (13%), oxidative stress (13%), osmoprotection (5%), photosynthesis (15%), protein stabilization (9%), and unclassified function (9%). Details of these proteins, including the relative spot intensities, are shown in Table 1.

## 4. Discussion

### 4.1. Proteins Related to Nitrogen Metabolism

Nitrogen metabolism often correlates with the nutritional status of field crops, and plants with higher nitrogen-use efficiency (NUE) normally have higher yields and protein contents [37]. Nitrogen limitation, as well as high temperature, affects all components related to NUE, including morphology of root systems, soil mineral uptake, and symbiotic nitrogen fixation [38]. Plants thus need to maintain nitrogen homeostasis within the body under such stressful conditions. In our study, proteins involved in nitrogen sensing and assimilation exhibited diverse expression patterns during treatments. One of the differentially-expressed proteins was identified as PII-like protein, an important signal transduction protein that senses and regulates N and C assimilation [39]. This (spot 6) increased in intensity with maximal upregulation in the N-efficient cultivar, possibly to regulate N levels under low N conditions for maintaining an optimal plant growth. In addition, two N-assimilating proteins, glutamine synthetase (spot 1) and ferredoxin-dependent glutamate synthase 2 (spot 11), exhibited a change in abundance. These enzymes catalyse two primary steps of the ammonia-assimilation pathway; the former accelerates the condensation of ammonia and glutamate to form glutamine, while the latter catalyses the synthesis of glutamate from glutamine and 2-oxoglutarate through transamidation reaction. Both of them were upregulated, probably to maintain N levels in leaves and achieve osmotic homeostasis via glutamate-based synthesis of proline. Our results are consistent with earlier works that showed increased level of these enzymes under low N [40,41,42] and drought [43] conditions. The overexpression of genes encoding these N-assimilating proteins has been associated with tolerance of plants under salinity [44] and drought stress [45]. Another N-regulatory protein, glucosamine-fructose-6-phosphate amino-transferase, which plays a key role in glutamine metabolic process and in the cell wall-chitin biosynthesis process, was upregulated (spot 28) in the stressed wheat plants.

### 4.2. Proteins Related to Photosynthesis

Photosynthesis is the key process that determines growth and development of plants, and encompasses reactions catalysed and regulated by proteins in the chloroplast. Four proteins related to photosynthesis varied in expression during the combined stress. One of them was identified as chlorophyll a/b binding protein, which confers stability to light-harvesting complexes (LHC) of photosystems. This protein (spot 34) increased in abundance under stress conditions, substantiating some earlier findings with reference to abiotic stresses [46,47]. The stress also affected the oxygen-evolving complex (OEC) involved in photo-oxidation rate of water. Photosystem II oxygen-evolving complex protein 1 (spot 5) increased, possibly to maintain stability of the complex. Parallel results were reported earlier in food crops, including rice [48], barley [49,50], wheat [50,51], and tomato [52] exposed to different abiotic stresses. Further, electron transport in chloroplast was also affected; spot intensity of FNR (spot 32), an enzyme that catalyses transfer of electrons from photosystem I (PSI) to ferredoxin, besides contributing to antioxidant defence, N fixation, and isoprenoid biosynthesis [53], was also increased. 

Combined stress of high temperature and low nitrogen affected both the abundance and mode of regulation of Rubisco, which catalyses CO_2_ fixation and is one of the primary determinants of photosynthetic rate. The decrease in its spot intensity (spots 40, 46, 52) could be due to its degradation. Our results are in line with some previous findings in wheat grown under high temperature [54] and drought conditions [55]. Environmental stress, particularly high temperature, not only degrades Rubisco, but also accelerates its inactivation by addition of inhibitory sugars to its active site. Moreover, Rubisco has a relatively low turnover number, compared with the other Calvin cycle enzymes. Activity of Rubisco is mainly regulated by a catalytic chaperone (Rubisco activase), which catalyses removal of inhibitory sugars from its active site, switching the enzyme to active mode [56]. In the present study, Rubisco activase increased significantly in abundance under stressful conditions signifying its potential to regulate Rubisco activity (spot 38). These results substantiate the earlier ones dealing with the effect of high temperature on rice [57] and *Carissa spinarum* [58], and of N deficiency on wheat [59]. 

### 4.3. Proteins Associated with Osmoregulation

One of the primary effects of high temperature is alteration of turgor within cells, often reducing plant growth and productivity. Plants synthesise compatible solutes that help in maintaining cell turgor, regulating redox homeostasis and stabilising proteins and other cell contents [60]. Proline functions in plants as an osmoprotectant, besides assisting in protein stabilization and buffering of redox potential [61]. It is synthesized mainly from glutamate by enzyme pyrroline-5-carboxylate synthetase, which catalyses conversion of glutamate to glutamic-γ-semialdehyde, which is reduced by pyrroline-5-carboxylate reductase to proline [62]. Pyroline-5-carboxylate synthetase enzyme was found to increase, implying accumulation of proline to decipher stress tolerance (spot 4). Histidine kinase (CRE1), a cytokinin receptor involved in cytokinin signalling and regarded as an osmosensor [63], was also upregulated (spot 8), possibly to confer osmotic balance during water-deficient conditions. Another protein with a role in osmoprotection [64] was identified as abscisic acid-inducible protein kinase (spot 16).

### 4.4. Proteins Related to Antioxidant Defense System

Overproduction of reactive oxygen species (ROS) due to abiotic stress can oxidize life-supporting biomolecules, such as lipids, proteins, carbohydrates, and nucleic acids. To regulate the oxidative homeostasis, plants possess a well-organised antioxidant defence system that helps in scavenging of ROS. Six differentially-expressed proteins, namely, superoxide dismutase (spot 35), ascorbate peroxidase (spot 15), glutathione transferase (spot 2), glyoxalase (spot 14), heme oxygenase (spot 10), and flavonol synthase (spot 20), were involved in antioxidant defence. Superoxide dismutase forms the first line of defence against oxidative stress by catalysing dismutation of superoxide ions to hydrogen peroxide. Ascorbate peroxidase scavenges H_2_O_2_ molecules formed by the action of SOD, and glutathione transferase catalyses conjugation of glutathione with toxic electrophiles generated as by-products of oxidative damage caused by hydroxyl radicals. Glutathione transferase helps in generating ascorbate from dehydroascorbate, besides safeguarding proteins from ROS-induced damage, by reducing hydroperoxides to less damaging alcohols [65]. Glyoxalase regulates glutathione homeostasis during abiotic stress [66], and is implicated in detoxification of methylglyoxal, a highly reactive dicarbonyl aldehyde compound, which accumulates in plants under stress as a glycolytic intermediate [67]. The fifth differentially expressed protein involved in oxidative defence was recognised as heme oxygenase, which also regulates various cellular processes, including iron acquisition and mobilization, stomatal opening and closure, and phytochrome chromophore synthesis [68]. In addition, flavonol synthase protein with a key role in biosynthesis of antioxidant flavonols [69] showed differential expression (spot 20). All these antioxidant proteins showed increase in spot intensity in the stressed material, compared to the control.

### 4.5. Proteins Related to Carbohydrate Metabolism

Starch degradation in plants serves as an effective means to tolerate stress by meeting energy demands and strengthening antioxidant defence system through production of NADPH, and by diverting substrates to oxidative pentose phosphate pathway [70]. In this study, three starch-degrading proteins, viz. sucrose phosphate synthase (spot 3), alpha-1,4-glucan phosphorylase (spot 7) and isoamylase (spot 42), increased in abundance due to stress. 

Moreover, combined stress affected the primary carbohydrate metabolic pathways, glycolysis, and tricarboxylic acid (TCA) cycle. Expression of two glycolytic enzymes; triosephosphate isomerase (spot 22) and glyceraldehyde 3-phosphate dehydrogenase (spot 31) decreased significantly. The former catalyses interconversion of dihydroxyacetone phosphate (DHAP) and d-glyceraldehyde 3-phosphate (GAP), while the latter facilitates conversion of glyceraldehyde 3-phosphate into 1,3-bisphosphoglycerate. Besides, two Krebs cycle enzymes, sedoheptulose-1,7-bisphosphatase (spot 27) and malate dehydrogenase (spot 61) were downregulated, possibly due to decreased CO_2_ uptake or as a strategy to save energy under stressful conditions [71]. 

### 4.6. Proteins Related to Energy Metabolism and Fatty Acid Metabolism

The combined stress induced overexpression of two proteins related to energy metabolism, viz. ATPase F1 α subunit (spot 12) and ATPase (spot 39). ATPase catalyses dissociation of ATP into ADP, and its upregulation in the stressed wheat plants possibly conferred energy expenditure required to cope with the stress.

Acetyl-CoA carboxylase catalyses carboxylation of acetyl-CoA to form malonyl-CoA during fatty acid synthesis. This cytosolic enzyme has a role in membrane stability and biosynthesis of flavonoids, which act as ROS scavengers [72]. The enzyme showed a significant increase (spot 37) in spot intensity, with expression levels relatively higher in N-efficient cultivar than in N-inefficient one. Our results go in line with earlier findings in peanut, where abundance of protein increased significantly under water-deficit conditions [73].

### 4.7. Proteins Related to Transcription and Protein Synthesis

Plant response to abiotic stress includes biosynthesis of tolerance-conferring proteins, and degradation or inactivation of unwanted or interfering ones. Transcription and protein synthesis are fine-tuned under stress. A marked decrease in transcription rate was evident from downregulation of RNA polymerase (spot 36). Other transcription-related proteins were histone acetyltransferase complex component (spot 44), SnRK1-interacting protein 1 (spot 47), zinc finger family protein (spot 51), and ABA (abscisic acid)-responsive element-binding protein 3 (spot 49), which help (i) in making DNA more accessible to transcription factors by reducing its interaction with histone proteins, (ii) in sensing low cellular glucose levels and enhancing metabolic signalling and carbon partitioning, (iii) in regulating transcription, protein–protein interaction and RNA binding, and (iv) in regulating ABA-dependent signalling systems that mediate stress adaptation. Spot intensity of all these transcription-related proteins markedly increased in stressed plants.

Abiotic stress inhibits translation in plants [74,75]. Intensity of five structural ribosomal proteins, viz. ribonucleoprotein A (spot 17), ribosomal protein L14 (spot 19), ribosomal protein S19 (spots 23 and 54), ribosomal protein S4 (spot 56), and ribosomal protein L23 (spot 60), including a translational factor 5A1 (spot 48), significantly declined in stressed plants, compared to the control. However, elongation factor-Tu (EF-Tu), the main protein synthesis elongation factor of plant organelles, which is also involved in chaperone activity, facilitation of protein renaturation, protein disulfide isomerase activity and degradation of N-terminally blocked proteins via the action of proteasome [76], was upregulated (spot 24). It has been shown that accumulation of EF-Tu in plants confers tolerance against environmental stress [76,77,78].

### 4.8. Changes in Proteins Involved in Protein Stabilization and Degradation

Most of the proteins are thermo-labile, and likely to get denatured under high temperature. Maintaining proteins in stable and functional conformations and preventing their aggregation are essential for survival of cells under high temperature. Various molecular chaperones are the basic components of innate immunity of plants. Five proteins; viz. disulfide isomerase (spot 9), chaperonin 60b (spot 13), cyclophilin (spot 18), heat-shock protein (spot 29), and heat shock-responsive transcription factor (spot 30), related to protein stabilization, were differentially expressed. Protein disulfide isomerase (PDI) is a chaperone engrossed in protein folding in endoplasmic reticulum via disulfide bond formation and isomerisation. Upregulation of PDI has been reported in bent grass and sorghum in response to drought stress [79,80]. Chaperonin 60b is another folding protein involved in protecting rubisco activase from denaturation and acclimating photosynthesis under heat stress [81]. Cyclophilins are ubiquitous proteins with an intrinsic peptidyl-prolyl cis-trans isomerase activity, and assist in protein folding and stability [82]. In the chloroplast, cyclophilins assist proteins such as oxygen-evolving complex proteins, to sequester and protect unassembled and degradation-prone lumenal proteins [83]. Two more proteins involved in protein stabilization were identified as small heat shock protein and heat shock-responsive transcription factor, which are involved mainly in heat tolerance. All these stabilising proteins were overexpressed, implying efficient protein repair systems and general protein stability that possibly facilitated plant survival under stressful conditions.

One of the efficient responses by plants to environmental stress is ubiquitination that involves alterations of proteome, including “switch on” of regulatory proteins, removal of malformed proteins and regulation of signalling proteins [84]. In this study, ubiquitin protein (spot 43) was upregulated in stressed plants, compared to the control.

### 4.9. Unclassified Proteins

During the period of stress, some proteins, the functions of which were neither clear nor directly related to stress conditions, were also expressed differentially. Osmotin-like protein (spot 21) is possibly involved in cell wall modifications. The functions of transposon protein, CACTA, En/Spm subclass (spot 45), and PR (Pathogenesis-related) -10 protein (spot 50) are still obscure. Inorganic pyrophosphatase family protein (spot 57) plays a key role in pyrophosphate metabolism, and regulates phosphorus levels within the cells. Methionine synthase (spot 59) regulates the synthesis of methionine, an amino-acid, which involves in the initiation of mRNA translation and regulation of molecular processes in the form of *S*-adenosylmethionine (SAM).

## 5. Conclusions

**Figure 5:**
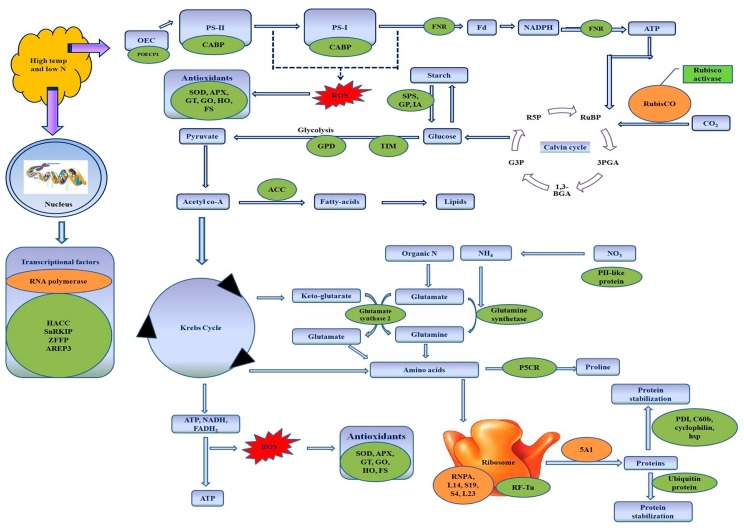
Schematic representation of differentially-expressed leaf proteins in wheat under combined stress of high temperature and low nitrogen. Proteins in green boxes were upregulated, whereas those in orange boxes were downregulated. OEC, oxygen evolving complex; POECP1, photosystem II oxygen-evolving complex protein 1; CABP, chlorophyll a/b binding protein; FNR, ferredoxin NADP reductase; Fd, ferredoxin; RuBP, ribulose-1,5-bisphosphate; 3PGA, 3-phosphoglycerate; 1,3-BGA, 1,3-bisphosphoglycerate; G3P, glyceraldehyde 3-phosphate; R5P, ribulose-5-phosphate; SPS, sucrose phosphate synthase ; GP, alpha-1,4-glucan phosphorylase; IA, isoamylase; TiM, triosephosphate isomerase; GPD, glyceraldehyde 3-phosphate dehydrogenase; SOD, superoxide dismutase; APX, ascorbate peroxidase; GT, glutathione transferase; GO, glyoxalase; HO, heme oxygenase; FS, flavonol synthase; ACC, acetyl-CoA carboxylase; P5CR, pyroline-5-caboxylate synthetase; PDI, protein disulfide isomerase; C60b, chaperonin 60b; hsp, heat shock protein; 5A1, translational factor 5A1; RNPA, ribonucleoprotein A; HACC, histone-acetyltransferase complex component; SnRKIP, SnRK1-interacting protein 1; ZFFP, zinc finger family protein; AREP3, ABA-responsive element-binding protein 3.

**Figure 6:**
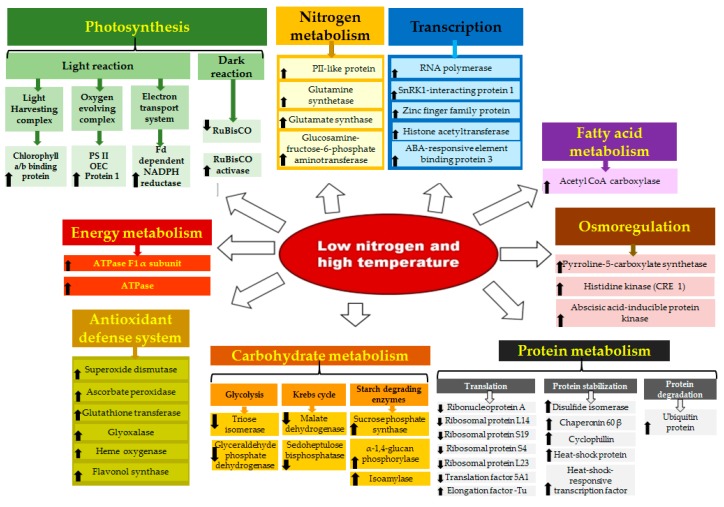
Representation and the associated processes of differentially-expressed leaf proteins in wheat under combined stress of high temperature and low nitrogen.

In conclusion, the combined stress of low N and high temperature alters different growth processes in wheat, the response of the plant being cultivar-dependent. Most of the proteins associated with defence mechanisms against heat, water deficit, N deficit, and oxidative stresses were upregulated; the degree of defence was higher in N-inefficient cultivar UP2382 than in N-efficient VL616. The majority of proteins involved in photosynthesis (RuBiSCo) and carbohydrate metabolism were downregulated with a more prominent setback in UP2382 than in VL616. On the basis of relevant biological function and increasing trend of expression, we could identify stress-responsive proteins (schematically represented in Figure 5 and Figure 6), which may be used as suitable candidates for increasing the tolerance of wheat under stress, and hence contributing to food security during harsh environmental conditions.

## Figures and Tables

**Table 1:** Identification, sub-cellular localization and quantitative analysis of differentially-expressed leaf proteins of nitrogen-efficient (VL616) and nitrogen-inefficient (UP2382) cultivars of wheat under the combined effect of high temperature (32 °C) and low nitrogen (4 mM).

S.N.	Accession No.	Name of Protein	Mr (Da)	Pi	M. SCO	No. of Matched Peptides	Location	Process	Mode of Regulation	Relative Spot Intensity	
										UP2382	VL616
1	Q45NB2	Glutamine synthetase	46,852	5.96	120	9	Chloroplast Mitochondria	Nitrogen metabolism	Upregulated	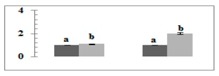	
2	P30110	Glutathione transferase	25,098	6.35	76	7	Cytosol	Oxidative stress	Upregulated	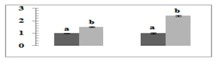	
3	Q6EZE7	Sucrose phosphate synthase	12,874	6.81	84	11	Cytosol	Carbohydrate metabolism	Upregulated	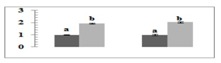	
4	M1NZ56	Pyrroline-5-carboxylate synthetase	30,883	5.74	122	6	Cytosol	Osmo-regulation	Upregulated	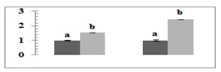	
5	P27665	Photosystem II oxygen-evolving complex protein 1	36,623	8.53	160	14	Chloroplast	Photosynthesis	Upregulated	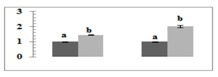	
6	Q6AUR2	PII-like protein	26,429	9.84	147	3	Chloroplast	Nitrogen metabolism	Upregulated	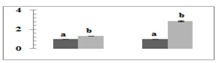	
7	D8L9G7	α-1,4-Glucan phosphorylase	89,520	7.20	134	7	Chloroplast	Osmo-regulation	Upregulated	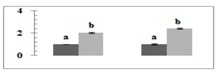	
8	Q66NG6	Plant histidine kinase (Cre 1)	120,729	6.22	102	8	Cytosol	Osmo-regulation	Upregulated	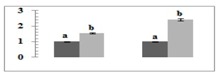	
9	P52589	Protein disulfide isomerase	56,921	5.01	149	3	ER	Protein stabilization	Upregulated	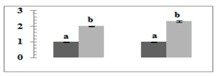	
10	G9BRR4	Heme oxygenase 4	32,415	5.81	62	6	Chloroplast	Oxidative stress	Upregulated	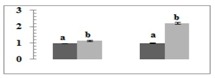	
11	M8CY84	Ferredoxin-dependent glutamate synthase 2	27,142	6.30	102	9	Chloroplast	Nitrogen metabolism	Upregulated	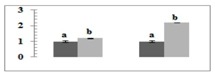	
12	P12112	ATPase F1 α subunit	48,233	6.27	134	6	Mitochondria	Energy metabolism	Upregulated	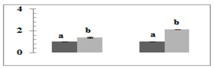	
13	P08823	Chaperonin 60 β precursor	54,321	4.61	158	5	Mitochondria	Protein stabilization	Upregulated	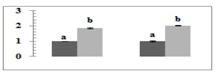	
14	Q8LD97	Glyoxylase I 7	32,564	5.82	74	2	Peroxisomes	Oxidative stress	Upregulated	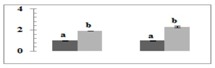	
15	M8B2C5	l-Ascorbate peroxidase 5	34,852	6.84	94	4	Chloroplast	Oxidative stress	Upregulated	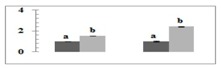	
16	M8A9B6	Abscisic acid-inducible protein kinase	37,023	5.51	54	6	Cytosol	Osmotic adjustment	Upregulated	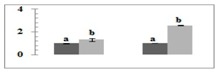	
17	Q43349	29 kDa ribonucleoprotein A, chloroplast precursor	28,326	4.75	165	5	Nucleus	Protein synthesis	Downregulated	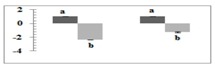	
18	P34791	Chloroplast-localised cyclophilin	26,527	8.48	146	6	Chloroplast	Protein stability	Upregulated	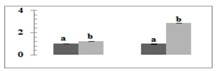	
19	Q95H51	Ribosomal protein L14	13,716	9.11	54	8	Ribosome	Protein synthesis	Downregulated	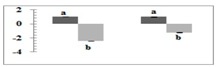	
20	X5CQE2	Flavonol synthase	36,401	5.33	94	8	Cytoplasm, Nucleus	Oxidative stress	Upregulated	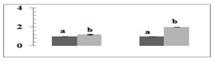	
21	M8BQR6	Osmotin like protein	27,148	7.87	135	6	Cell wall	Cell wall synthesis	Upregulated	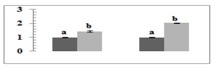	
22	M7Z1M4	Triosephosphate isomerase	31,955	6.00	148	14	Chloroplast, Cytosol	Glycolysis	Downregulated	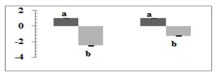	
23	P60577	Ribosomal protein S19	10,589	11.0	50	15	Ribosome, Chloroplast, Mitochondria	Protein synthesis	Downregulated	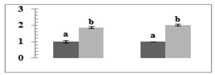	
24	A0A1D5ZWW7	Translation elongation factor-Tu	52,275	5.09	99	9	Plastid, Mitochondria, Cytosol	Protein synthesis	Upregulated	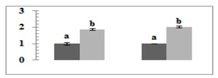	
25	--	Unknown	9,371	6.25	58	3	-	-	Upregulated	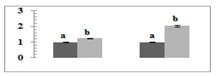	
26	--	Predicted protein	6,649	4.62	42	2	-	-	Downregulated	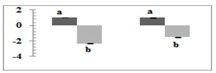	
27	P46285	Sedoheptulose-1,7-bisphosphatase	42,068	6.26	153	15	Chloroplast	Calvin cycle	Downregulated	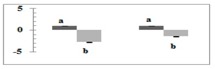	
28	M7ZTK2	Glucosamine-fructose-6-phosphate aminotransferase	73,834	6.98	47	6	Cytosol	Nitrogen metabolism	Upregulated	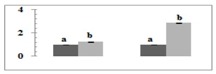	
29	Q9ZSR6	Small heat shock protein	25,622	9.20	61	7	Nucleus	Protein stabilization	Upregulated	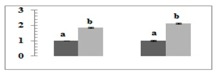	
30	C1K2Q2	Heat shock responsive transcription factor	38,362	5.21	54	5	Nucleus	Protein stabilization	Upregulated	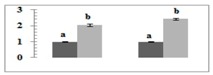	
31	A5YVV3	Glyceraldehyde 3-phosphate dehydrogenase	42,766	7.62	141	4	Plastid, Mitochondria	Glycolysis	Downregulated	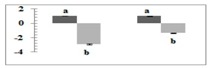	
32	Q9FKW6	RecName: Full=Ferredoxin--NADP reductase, chloroplastic	41,322	8.54	139	7	Chloroplast	Photosynthesis	Upregulated	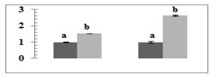	
33	P11383	Ribulose-1,5-bisphosphate carboxylase/oxygenase large subunit	53,901	6.22	351	18	Chloroplast	Photosynthesis	Downregulated	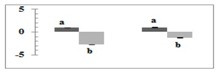	
34	W5G9W6	Chlorophyll a-b binding protein 1	24,836	5.11	112	6	Chloroplast	Photosynthesis	Upregulated	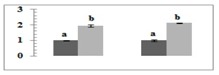	
35	A0A1D5TQL0	Cu/Zn superoxide dismutase	20,352	5.35	130	4	Cytosol	Oxidative stress	Upregulated	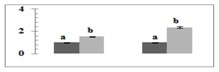	
36	Q9XPS8	RNA polymerase β chain	79,908	9.00	217	8	Nucleus	Transcription	Downregulated	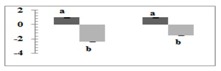	
37	P56765	Acetyl-CoA carboxylase β subunit	40,207	9.02	53	5	Cytoplasm, plastid	Fatty acid metabolism	Upregulated	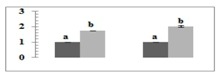	
38	P10896	Rubisco activase chloroplast precursor	51,786	5.43	138	4	Chloroplast	Photosynthesis	Upregulated	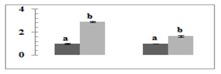	
39	P83970	ATPase	110,868	6.50	169	9	Plasma membrane, tonoplast	Energy metabolism	Upregulated	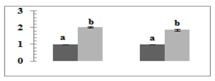	
40	P11383	Ribulose-1,5-bisphosphate carboxylase/oxygenase small subunit	52,235	6.41	45	8	Chloroplast	Photosynthesis	Downregulated	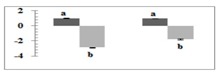	
41	A5BMQ9	Hypothetical protein VITISV_041859	50,670	6.25	38	5	-	-	Downregulated	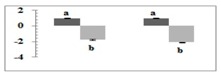	
42	D0TZF0	Isoamylase precursor	75,052	6.40	52	2	Cytosol	Carbohydrate metabolism	Upregulated	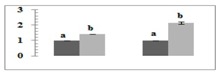	
43	P69326	Ubiquitin	4,693	8.90	72	6	Cytosol	Protein degradation	Upregulated	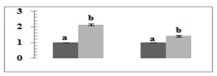	
44	W5B4J0	Histone acetyl-transferase complex component	16,392	5.41	48	4	Nucleus	Transcription	Upregulated	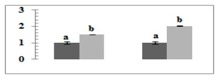	
45	Q2QWY8	Transposon protein, putative, CACTA, En/Spm sub-class	55,850	8.34	41	7	Nucleus	-	Upregulated	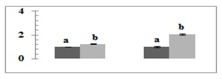	
46	Q9SAX6	Ribulose-1,5-bisphosphate carboxylase/oxygenase small subunit, partial	13,215	6.60	87	10	Chloroplast	Photosynthesis	Downregulated	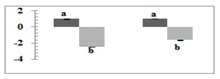	
47	B6TJX4	SnRK1-interacting protein 1	14,800	7.23	82	6	Nucleus	Transcription	Upregulated	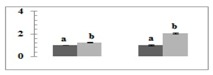	
48	M7ZIN5	Translation factor 5A1	17,532	5.70	114	5	Ribosome	Protein synthesis	Downregulated	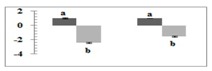	
49	D7LVK3	ABA-responsive element binding protein 3	32,674	6.53	43	9	Nucleus	Transcription	Upregulated	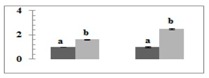	
50	B5B3P8	PR-10 protein	16,464	7.04	22	8	Nucleus	-	Downregulated	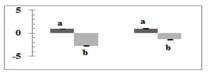	
51	A0A1D5WC57	Zinc finger family protein	38,647	8.01	47	9	Nucleus	Transcription	Upregulated	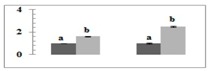	
52	W5ZRX4	Ribulose-1,5-bisphosphate carboxylase/oxygenase large subunit	52,563	6.05	212	5	Chloroplast	Photosynthesis	Downregulated	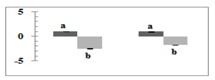	
53	--	Predicted protein	11,607	10.46	125	7	-	-	Upregulated	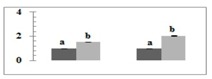	
54	S4Z9E7	Ribosomal protein S19	6,795	9.12	110	6	Ribosome	Protein synthesis	Downregulated	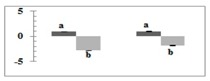	
55	--	Unnamed protein product	4,414	7.90	24	8	-	-	Downregulated	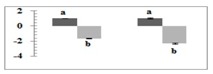	
56	Q95H61	Ribosomal protein S4	23,540	10.80	47	6	Ribosome	Protein synthesis	Downregulated	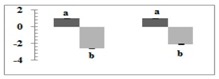	
57	Q9LXC9	Inorganic pyrophosphatase family protein	32,567	5.72	112	10	Chloroplast	Phosphorus metabolism	Upregulated	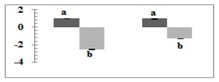	
58	A2ZLN9	Hypothetical protein OsI_38734	15,336	4.25	41	11	-	Chloroplast	Upregulated	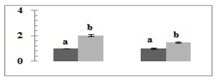	
59	A0A1D5ZI60	Methionine synthase	54,794	5.79	101	8	Mitochondria	Amino-acid metabolism	Upregulated	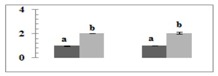	
60	P69667	Ribosomal protein L23	9,563	10.02	71	6	Ribosome	Protein synthesis	Downregulated	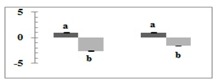	
61	A0A1D5ST37	Malate dehydrogenase	38,104	5.61	58	8	Mitochondria	Citric acid cycle	Downregulated	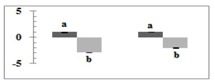	

S.N. = Spot Number, Mr = Molecular Weight; Pi = Isoelectric Point; M.SCO = Mascot Score. Dark bars indicate control condition (ambient temperature and optimum N level) and light bars indicate treatment condition (low N and high temperature). Spot volumes were analysed by PDQuest software. The fold change of up-regulated protein spot volumes was calculated by treatment/control, whereas the change fold of down-regulated protein spot volumes was calculated by control/treatment. Bars with different letters are significantly different from each other in a single cultivar across treatments (Duncan’s post-test). PR= Pathogenesis-related; ABA = abscisic acid.

## References

[1] Fischer R., Edmeades G.O. (2010). Breeding and cereal yield progress. Crop Sci..

[2] Zou J., Liu C., Chen X. (2011). Proteomics of rice in response to heat stress and advances in genetic engineering for heat tolerance in rice. Plant Cell Rep..

[3] Madan P., Jagadish S.V., Craufurd P.Q., Fitzgerald M., Lafarge T., Wheeler T.R. (2012). Effect of elevated CO_2_ and high temperature on seed-set and grain quality of rice. J. Exp. Bot..

[4] Sánchez B., Rasmussen A., Porter J.R. (2014). Temperatures and the growth and development of maize and rice, a review. Glob. Chang. Biol..

[5] Intergovernmental Panel on Climate Change (2007). Climate Change 2007: The Physical Science Basis.

[6] Intergovernmental Panel on Climate Change (2014). Climate Change 2014: Impacts, Adaptation, and Vulnerability. Part A, Global and Sectoral Aspects.

[7] Bita C.E., Gerats T. (2013). Plant tolerance to high temperature in a changing environment, scientific fundamentals and production of heat stress-tolerant crops. Front. Plant Sci..

[8] Glass A.D.M. (2003). Nitrogen use efficiency of crop plants, physiological constraints upon nitrogen absorption. Crit. Rev. Plant Sci..

[9] Good A.G., Shrawat A.K., Muench D.G. (2004). Can less yield more? Is reducing nutrient input into the environment compatible with maintaining crop production?. Trends Plant Sci..

[10] Reynolds M., Bonnett D., Chapman S.C., Furbank R.T., Manes Y., Mather D.E., Parry M.A.J. (2011). Raising yield potential of wheat. I. Overview of a consortium approach and breeding strategies. J. Exp. Bot..

[11] Parry M.A.J., Hawkesford M.J. (2012). An integrated approach to crop genetic improvement. J. Integr. Plant Biol..

[12] Hirayama T., Shinozaki K. (2010). Research on plant abiotic stress responses in the post-genome era, past, present and future. Plant J..

[13] Chew Y.H., Halliday K.J. (2011). A stress-free walk from *Arabidopsis* to crops. Curr. Opin. Biotechnol..

[14] Ahuja I., de Vos R.C.H., Bones A.M., Hall R.D. (2010). Plant molecular stress responses face climate change. Trends Plant Sci..

[15] Mitler R., Finka A., Goloubinoff P. (2012). How do plants feel the heat?. Trends Biochem. Sci..

[16] Yousuf P.Y., Ahmad A., Hemant Ganie A.H., Iqbal M. (2015). Potassium and calcium application ameliorates growth and oxidative homeostasis in salt-stressed Indian mustard plants. Pak. J. Bot..

[17] Yousuf P.Y., Ahmad A., Aref I.M., Ozturk M., Hemant Ganie A.H., Iqbal M. (2016). Salt-stress-responsive chloroplast proteins in *Brassica juncea* genotypes with contrasting salt tolerance and their quantitative PCR analysis. Protoplasma.

[18] Larkindale J., Vierling E. (2008). Core genome responses involved in acclimation to high temperature. Plant Physiol..

[19] Jamil A., Riaz S., Ashraf M., Foolad M.R. (2011). Gene expression profiling of plants under salt stress. Crit. Rev. Plant Sci..

[20] Li H., Payne W.A., Michels G.J., Charles M.R. (2008). Reducing plant abiotic and biotic stress, drought and attacks of greenbugs, corn leaf aphids and virus disease in dryland sorghum. Environ. Exp. Bot..

[21] Yousuf P.Y., Ahmad A., Ganie A.H., Iqbal M. (2016). Salt stress-induced modulations in the shoot proteome of *Brassica juncea* genotypes. Environ. Sci. Pollut. Res..

[22] Leff B., Ramankutty N., Foley J.A. (2004). Geographic distribution of major crops across the world. Glob. Biogeochem. Cycles.

[23] Food and Agriculture Organization (2012). FAOSTAT, Food Balance Sheets. faostat.fao.org.

[24] Gourdji S.M., Sibley A.M., Lobell D.B. (2013). Global crop exposure to critical high temperatures in the reproductive period, historical trends and future projections. Environ. Res. Lett..

[25] Koehler A.K., Challinor A.J., Hawkins E., Asseng S. (2013). Influences of increasing temperature on Indian wheat, quantifying limits to predictability. Environ. Res. Lett..

[26] Teixeira E.I., Fischer G., van Velthuizen H., Walter C., Ewert F. (2013). Global hot-spots of heat stress on agricultural crops due to climate change. Agric. For. Meteorol..

[27] Kharel T.P., Clay D.E., Clay S.A., Beck D., Reese C., Carlson G., Park H. (2011). Nitrogen and water stress affect winter wheat yield and dough quality. Agron. J..

[28] Asseng S., Ewert F., Martre P., Rötter R.P., Lobell D.B., Cammarano D., Kimball B.A., Ottman M.J., Wall G.W., White J.W. (2015). Rising temperatures reduce global wheat production. Nat. Clim. Chang..

[29] Tjoelker M.G., Reich P.B., Oleksyn J. (1999). Changes in leaf nitrogen and carbohydrates underlie temperature and CO_2_ acclimation of dark respiration in five boreal tree species. Plant Cell Environ..

[30] Semenov M.A., Jamieson P.D., Martre P. (2007). Deconvoluting nitrogen use efficiency in wheat: A simulation study. Eur. J. Agron..

[31] Chandna R., Kaur G., Iqbal M., Khan I., Ahmad A. (2012). Differential response of wheat genotypes to applied nitrogen, biochemical and molecular analysis. Arch. Agron. Soil Sci..

[32] Isaacson T., Damasceno C.M., Saravanan R.S., He Y., Catala C., Saladie M., Rose J.K. (2006). Sample extraction techniques for enhanced proteomic analysis of plant tissues. Nat. Protoc..

[33] O’Farrel P.H. (1975). High resolution two-dimensional electrophoresis of proteins. J. Biol. Chem..

[34] National Center for Biotechnology Information (NCBI). https://www.ncbi.nlm.nih.gov.

[35] The UniProt Consortium (2017). UniProt: The universal protein knowledgebase. Nucleic Acids Res..

[36] Guda C. (2006). pTARGET: A web server for predicting protein subcellular localization. Nucleic Acids Res..

[37] Yue S., Meng Q., Zhao R., Li F., Chen X., Zhang F., Cui Z. (2012). Critical nitrogen dilution curve for optimizing nitrogen management of winter wheat production in the north China plain. Agron. J..

[38] Christophe S., Jean-Christophe A., Annabelle L., Alain O., Marion P., Anne-Sophie V., Shanker A., Venkateswarlu B. (2011). Plant N fluxes and modulation by nitrogen, heat and water stresses: A review based on comparison of legumes and non-legume plants. Abiotic Stress in Plants—Mechanisms and Adaptations.

[39] Huergo L.F., Chandra G., Merrick M. (2013). PII signal transduction proteins, nitrogen regulation and beyond. FEMS Microbiol. Rev..

[40] Kichey T., Le Gouis J., Sangwan B., Hirel B., Dubois F. (2005). Changes in the cellular and subcellular localization of glutamine synthetase and glutamate dehydrogenase during flag leaf senescence in wheat (*Triticum aestivum* L.). Plant Cell Physiol..

[41] Lemaitre T., Gaufichon L., Boutet-Mercey S., Christ A., Masclaux-Daubresse C. (2008). Enzymatic and metabolic diagnostic of nitrogen deficiency in *Arabidopsis thaliana* Wassileskija accession. Plant Cell Physiol..

[42] Kant S., Bi Y., Rothstein S.J. (2011). Understanding plant response to nitrogen limitation for the improvement of crop nitrogen use efficiency. J. Exp. Bot..

[43] Aranjuelo I., Molero G., Erice G., Avice J.C., Nogues S. (2011). Plant physiology and proteomics reveals the leaf response to drought in alfalfa (*Medicago sativa* L.). J. Exp. Bot..

[44] Hoshida H., Tanaka Y., Hibino T., Hayashi Y., Tanaka A., Takabe T., Takabe T. (2000). Enhanced tolerance to salt stress in transgenic rice that overexpresses chloroplast glutamine synthetase. Plant Mol. Biol..

[45] Pascual M.B., Jing Z.P., Kirby E.G., Canovas F.M., Gallardo F. (2008). Response of transgenic poplar overexpressing cytosolic glutamine synthetase to phosphinothricin. Phytochemistry.

[46] Xu Y.H., Liu R., Yan L., Liu Z.Q., Jiang S.C., Shen Y.Y., Wang X.F., Zhang D.P. (2012). Light-harvesting chlorophyll a/b-binding proteins are required for stomatal response to abscisic acid in *Arabidopsis*. J. Exp. Bot..

[47] Yousuf P.Y., Ahmad A., Ganie A.H., Sareer O., Krishnapriya V., Aref I.M., Iqbal M. (2016). Antioxidant response and proteomic modulations in Indian mustard grown under salt stress. Plant Growth Regul..

[48] Yan S., Tang Z., Su W., Sun W. (2005). Proteomic analysis of salt stress-responsive proteins in rice root. Proteomics.

[49] Rollins J.A., Habte E., Templer S.E., Colby T., Schmidt J., von Korff M. (2013). Leaf proteome alterations in the context of physiological and morphological responses to drought and heat stress in barley (*Hordeum vulgare* L.). J. Exp. Bot..

[50] Kosova K., Vitamvas P., Prasil I.T. (2014). Proteomics of stress responses in wheat and barley—search for potential protein markers of stress tolerance. Front. Plant Sci..

[51] Wang M.C., Peng Z.Y., Li C.L., Li F., Liu C., Xia G.M. (2008). Proteomic analysis on a high salt tolerance introgression strain of *Triticum aestivum*/*Thinopyrum ponticum*. Proteomics.

[52] Zhou S.P., Sauve R.J., Liu Z., Reddy S., Bhatti S., Hucko S.D., Fish T., Thannhauser T.W. (2011). Identification of salt-induced changes in leaf and root proteomes of the wild tomato, *Solanum chilense*. J. Am. Soc. Hortic. Sci..

[53] Hanke G.T., Okutani S., Satomi Y., Takao T., Suzuki A., Hase T. (2005). Multiple iso-proteins of FNR in *Arabidopsis*, evidence for different contributions to chloroplast function and nitrogen assimilation. Plant Cell Environ..

[54] Chauhan H., Khurana N., Tyagi A.K., Khurana J.P., Khurana P. (2011). Identification and characterization of high temperature stress responsive genes in bread wheat (*Triticum aestivum* L.) and their regulation at various stages of development. Plant Mol. Biol..

[55] Singh B., Usha K. (2003). Salicylic acid induced physiological and biochemical changes in wheat seedlings under water stress. Plant Growth Regul..

[56] Portis A.R. (2003). Rubisco activase—Rubisco’s catalytic chaperone. Photosynth. Res..

[57] Lee D.G., Ahsan N., Lee S.H., Kang K.Y., Bahk J.D., Lee I.J., Lee B.H. (2007). A proteomic approach in analyzing heat-responsive proteins in rice leaves. Proteomics.

[58] Zhang M., Li G., Huang W., Bi T., Chen G., Tang Z., Su W., Sun W. (2010). Proteomic study of *Carissa spinarum* in response to combined heat and drought stress. Proteomics.

[59] Bahrman N., Le Gouis J., Negroni L., Amilhat L., Leroy P., Laine A.L., Jaminon O. (2004). Differential protein expression assessed by two-dimensional gel electrophoresis for two wheat varieties grown at four nitrogen levels. Proteomics.

[60] Rivero R.M., Mestre T.C., Mittler R., Rubio F., Garcia-Sanchez F., Martinez V. (2014). The combined effect of salinity and heat reveals a specific physiological, biochemical and molecular response in tomato plants. Plant Cell Environ..

[61] Suzuki N., Koussevitzky S., Mittler R., Miller G. (2012). ROS and redox signalling in the response of plants to abiotic stress. Plant Cell Environ..

[62] Murahama M., Yoshida T., Hayashi F., Ichino T., Sanada Y., Wada K. (2001). Purification and characterization of δ1-pyrroline-5-carboxylate reductase isoenzymes, indicating differential distribution in spinach (*Spinacia oleracea* L.) leaves. Plant Cell Physiol..

[63] Inoue T., Higuchi M., Hashimoto Y., Seki M., Kobayashi M., Kato T., Tabata S., Shinozaki K., Kakimoto T. (2001). Identification of CRE1 as a cytokinin receptor from *Arabidopsis*. Nature.

[64] Anderberg R.J., Walker-Simmons M.K. (1992). Isolation of a wheat cDNA clone for an abscisic acid-inducible transcript with homology to protein kinases. Proc. Natl. Acad. Sci. USA.

[65] Dixon D.P., Edwards R. (2010). Glutathione Transferases. Arabidopsis Book.

[66] Hossain M.A., Hasanuzzaman M., Fujita M. (2011). Coordinate induction of antioxidant defense and glyoxalase system by exogenous proline and glycine betaine is correlated with salt tolerance in mung bean. Front. Agric. China.

[67] Yadav S.K., Singla-Pareek S.L., Ray M., Reddy M.K., Sopory S.K. (2005). Methylglyoxal levels in plants under salinity stress are dependent on glyoxalase I and glutathione. Biochem. Biophys. Res. Commun..

[68] Shekhawat G.S., Verma K. (2010). Heme oxygenase (HO), an overlooked enzyme of plant metabolism and defence. J. Exp. Bot..

[69] Fini A., Brunetti C., Di Ferdinando M., Ferrini F., Tattini M. (2011). Stress-induced flavonoid biosynthesis and the antioxidant machinery of plants. Plant Signal Behav..

[70] Lloyd J.R., Kossmann J., Ritte G. (2005). Leaf starch degradation comes out of the shadows. Trends Plant Sci..

[71] Sobhanian H., Aghaei K., Komatsu S. (2011). Changes in the plant proteome resulting from salt stress, toward the creation of salt-tolerant crops?. J. Proteom..

[72] Figueroa-Balderas R.E., Garcia-Ponce B., Rocha-Sosa M. (2006). Hormonal and stress induction of the gene encoding common bean acetyl-coenzyme A carboxylase. Plant Physiol..

[73] Kottapalli K.R., Rakwal R., Shibato J., Burow G., Tissues D., Burke J., Puppala N., Burow M., Payton P. (2009). Physiology and proteomics of the water-deficit stress response in three contrasting peanut genotypes. Plant Cell Environ..

[74] Matsuura H., Kiyotaka U., Ishibashi Y., Yamaguchi M., Hirata K., Demura T., Kato K. (2010). A short period of mannitol stress but not LiCl stress led to global translational repression in plants. Biosci. Biotechnol. Biochem..

[75] Sormani R., Delannoy E., Lageix S., Bitton F., Lanet E., Saez-Vasquez J., Deragon J.M., Renou J.P., Robaglia C. (2011). Sublethal cadmium intoxication in *Arabidopsis thaliana* impacts translation at multiple levels. Plant Cell Physiol..

[76] Fu J., Momcilovic I., Prasad P.V. (2012). Roles of protein synthesis elongation factor EF-Tu in heat tolerance in plants. J. Bot..

[77] Bhadula S.K., Elthon T.E., Habben J.E., Helentjaris T.G., Jiao S., Ristic Z. (2001). Heat-stress induced synthesis of chloroplast protein synthesis elongation factor (EF-Tu) in a heat-tolerant maize line. Planta.

[78] Bukovnik U., Fu J., Bennett M., Prasad P.V.V., Ristic Z. (2009). Heat tolerance and expression of protein synthesis elongation factors, EF-Tu and EF-1α, in spring wheat. Funct. Plant Biol..

[79] Merewitz E.B., Gianfagna T., Huang B. (2011). Protein accumulation in leaves and roots associated with improved drought tolerance in creeping bentgrass expressing an ipt gene for cytokinin synthesis. J. Exp. Bot..

[80] Jedmowski C., Ashoub A., Beckhaus T., Berberich T., Karas M., Brüggemann W. (2014). Comparative analysis of *Sorghum bicolor* proteome in response to drought stress and following recovery. Int. J. Proteom..

[81] Salvucci M.E. (2008). Association of Rubisco activase with chaperonin-60β, a possible mechanism for protecting photosynthesis during heat stress. J. Exp. Bot..

[82] Trivedi D.K., Ansari M.W., Tuteja N. (2013). Multiple abiotic stress responsive rice cyclophilin (OsCYP-25) mediates a wide range of cellular responses. Commun. Integr. Biol..

[83] Romano P., Horton P., Gray J.E. (2004). The *Arabidopsis thaliana* cyclophilin gene family. Plant Physiol..

[84] Stone S.L. (2014). The role of ubiquitin and the 26S proteasome in plant abiotic stress signaling. Front. Plant Sci..

